# Effect of Subcutaneous Insulin on Spirometric Maneuvers in Patients with Type 1 Diabetes: A Case-Control Study

**DOI:** 10.3390/jcm9051249

**Published:** 2020-04-25

**Authors:** Enric Sánchez, Chadia Mizab, Ariadna Sauret, Ferran Barbé, Raquel Martí, Carolina López-Cano, Marta Hernández, Liliana Gutiérrez-Carrasquilla, Paola Carmona, Jessica González, Mireia Dalmases, Cristina Hernández, Rafael Simó, Albert Lecube

**Affiliations:** 1 Endocrinology and Nutrition Department, University Hospital Arnau de Vilanova, Obesity, Diabetes and Metabolism (ODIM) Research Group, Institut de Recerca Biomèdica de Lleida (IRBLleida), University of Lleida, 25198 Lleida, Spain; esanchez@irblleida.cat (E.S.); chadia.mizab@gmail.com (C.M.); ariadnags973@gmail.com (A.S.); rmarti@irblleida.cat (R.M.); karolopezc@gmail.com (C.L.-C.); martahernandezg@gmail.com (M.H.); liligutierrezc@gmail.com (L.G.-C.); 2 Respiratory Department, University Hospital Arnau de Vilanova-Santa María, Translational Research in Respiratory Medicine, IRBLleida, University of Lleida, 25198 Lleida, Spain; febarbe.lleida.ics@gencat.cat (F.B.); pcarmona@irblleida.cat (P.C.); jegonzalez.lleida.ics@gencat.cat (J.G.); mdalmases.lleida.ics@gencat.cat (M.D.); 3 Centro de Investigación Biomédica en Red de Enfermedades Respiratorias (CIBERES), Instituto de Salud Carlos III (ISCIII), 28029 Madrid, Spain; 4 Endocrinology and Nutrition Department, University Hospital Vall d’Hebron, Diabetes and Metabolism Research Unit, Vall d’Hebron Institut de Recerca (VHIR), Autonomous University of Barcelona, 08035 Barcelona, Spain; cristina.hernandez@vhir.org; 5 Centro de Investigación Biomédica en Red de Diabetes y Enfermedades Metabólicas Asociadas (CIBERDEM), Instituto de Salud Carlos III (ISCIII), 28029 Madrid, Spain

**Keywords:** basal insulin, bolus insulin, lung function, spirometry, type 1 diabetes

## Abstract

In order to compare spirometric maneuvers in adults according to the presence of type 1 diabetes, a case-control study including 75 patients with type 1 diabetes and 75 controls matched by sex, age, and body mass index were designed. In addition, 75 patients with type 1 diabetes were added to examine the potential the impact of subcutaneous insulin therapy on pulmonary function. Lung function measurements were assessed according to the global initiative for chronic obstructive lung disease guidelines. Basal insulin included long-acting insulin analogues and the delivered background insulin in patients with pump therapy. Bolus insulin included rapid-acting insulin analogues and the delivered insulin to cover postprandial hyperglycemias. Patients with type 1 diabetes showed lower spirometric values in comparison to the control group, together with a higher prevalence of forced expiratory volume in the first second (FEV1) <80% (10.7% vs. 2.7%, *p* = 0.044) and restrictive ventilatory pattern (10.7% vs. 0%, *p* = 0.006) The dose of basal insulin (U/kg/day) showed a negative correlation with forced vital capacity (FVC) (*r* = −0.205, *p* = 0.012) and FEV1 (*r* = −0.182, *p* = 0.026). The optimal cut-off value for identifying patients with a restrictive spirometric pattern was 0.5 U/kg/day of basal insulin. Additionally, basal insulin (U/kg/day) independently predicted the presence of both a restrictive spirometric pattern (OR = 77.1 (3.2 to 1816.6), *p* = 0.007) and an abnormal FEV1 (OR = 29.9 (1.5 to 562.8), *p* = 0.023). In patients with type 1 diabetes, higher basal insulin dosage seems to be related with an impairment of pulmonary function.

## 1. Introduction

An increasing amount of evidence has been published during the past decade pointing to the deleterious effect of type 2 diabetes on pulmonary function [1]. Despite the fact that it seems unlikely for the lungs to be affected by diabetes, their great vascularization and richness in collagen and elastin fibers make the pulmonary parenchyma a potential target for the diabetes milieu [2]. In truth, the lung is affected by the same histological and physiological disturbances associated with diabetes in other organ systems, such as the thickening of the capillary walls and the alveolar basal membrane [3]. Moreover, lung dysfunction appears more frequently in subjects with poorer metabolic control and who have suffered a longer duration of the disease [4,5]. However, data related to pulmonary function in adult population with type 1 diabetes is still scarce.

Previous studies investigating lung function in patients with type 1 diabetes have been performed on small cohorts, and mainly focused on children and adolescents [6,7]. Although some of them reported normal results [6], the impairment in pulmonary function was recurrently described in this population [7]. Some of the suggested mechanisms underlying lung involvement in type 2 diabetes, such as microangiopathic changes and non-enzymatic glycosylation of tissue proteins are also shared by type 1 diabetes and primarily associated with a restrictive pulmonary pattern [1,8,9]. The alarming increase in the prevalence of obesity among patients with type 1 diabetes also favors the negative impact of insulin resistance and low-grade inflammation on the growth and metabolism of bronchoalveolar epithelium and vascular smooth muscle [1,10]. In fact, hyperinsulinemia increases the proliferation of primary human airway smooth muscle cells and its hyperresponsiveness and contractility upon insulin exposure, and consequently, this association has been proposed as a potential explanation for the positive correlation between incidence of type 1 diabetes and wheezing [11]. Furthermore, small reductions in pulmonary function parameters such as forced expiratory volume in the first second (FEV1) have been shown when insulin is delivered by inhalation, although this effect is not progressive over time and disappears when the treatment is ceased [12,13,14]. However, no studies have been conducted to determine whether subcutaneous insulin therapy per se is an independent contributing factor for the reduced pulmonary function described in type 1 diabetes. Therefore, we have designed a case-control study comparing spirometric maneuvers in adult individuals according to the presence of type 1 diabetes closely matching the most important variables affecting lung function. Moreover, we have also targeted the potential impact of the characteristics of insulin therapy on pulmonary function.

## 2. Materials and Methods

### 2.1. Statement on Ethics

A written informed consent was obtained from all participants and the study was conducted according to the ethical guidelines of the Helsinki Declaration. The human ethics committee from the University Hospital Arnau de Vilanova approved the study (CEIC-1516).

### 2.2. Design of the Study and Description of the Study Population

In this study we have assessed the influence of type 1 diabetes in lung function following the *Strengthening the Reporting of Observational Studies in Epidemiology (STROBE)* guidelines for reporting case–control studies [15]. As changes in FEV1 (% of predicted) were our main variable of interest, we used the following formula for the sample size calculation: *n* = (2 × (Zα + Z*β*)^2^ × *s*^2^)/*d*^2^**,** where the alpha level for a two-tailed test was set at *p* < 0.05 (*Z*α) and the minimum acceptable power level was considered to be 0.90 (*Zβ*), 15.6 was the standard deviation of FEV1 detected in a previous study with adult patients with type 1 diabetes (s) [16], 10% was postulated as a clinically significant difference in FEV1 between the two groups (*d*) and *n* is the sample size for each group. Therefore, *n* = (2 × (1.96 + 1.28)^2^ × 15.6^2^)/10^2^ = 62.2.

The study examined a total of 236 Caucasian subjects with type 1 diabetes when attending the outpatient Diabetes Clinic from June 2016 to June 2019 (Supplementary Figure S1). The inclusion criteria were as follows: Type 1 diabetes with at least three years of follow-up, age between 18 and 70 years old, a BMI lower than 30 kg/m^2^ and no medical history of chronic pulmonary disease or asthma. Among the 177 patients who met the inclusion criteria, we excluded 27 for the following reasons: Unwillingness to participate in the study (*n* = 8), concomitant treatment with corticosteroids (*n* = 3), an inability to perform the spirometric maneuvers correctly (*n* = 2), pregnancy (*n* = 12), and heart failure (*n* = 2). Finally, spirometry was performed in 150 subjects under treatment both with multiple daily injections regimens (88%) or insulin pump therapy (12%).

Basal insulin included long-acting insulin analogues (glargine 100 units/mL, glargine 300 units/mL and degludec) and the delivered background insulin in patients with pump therapy. Bolus insulin included rapid-acting insulin analogues (aspart, lispro, and glulisine) and the delivered insulin to cover the increase in blood sugar from meals. Insulin dose was expressed as daily units per kilogram of body weight (U/kg/day).

Diabetic retinopathy was diagnosed when a fundus examination performed by indirect ophthalmoscopy or fundus photographs within at least two years before inclusion revealed microvascular abnormalities. Nephropathy was diagnosed when patients presented at least two determinations of the urinary albumin-to-creatinine ratio > 300 mg/g.

On this basis, 75 patients with type 1 diabetes were recruited for the case-control study. We aimed to select one control for every case. Subsequently, 75 healthy subjects without type 1 diabetes or pulmonary disease were recruited from June 2018 to July 2019 among the employees of our institution and relatives of patients with diabetes. Controls were individually matched to cases by sex, age (within 3-years range), and BMI (within 2.0 kg/m^2^ range). The main clinical features of the case-control study population are displayed in Table 1. Routine laboratory tests were done to evaluate fasting plasma glucose (FPG) and glycosylated hemoglobin (HbA1c). History of smoking habit (non-smoker/current/former smoker) was recorded. Smokers who stopped smoking ≥1 year prior to recruitment were considered former smokers. The Bonora equation [17] was used to estimate visceral adipose tissue and the Hume equation was used to estimate lean body mass [18].

Following this 1:1 case-control study, and to assess the influence of insulin therapy on lung function of patients with type 1 diabetes, we extended this group to a total of 150 subjects with type 1 diabetes. The main clinical features of the study population are displayed in Table 2.

### 2.3. Measurement of Lung Function

Forced spirometry was executed using a portable ultrasonic spirometer (Datospir©, Sibelmed, Barcelona, Spain). Lung function tests were performed in accordance with the American Thoracic Society and European Respiratory Society Guidelines [19]. Participants were required to complete at least three reproducible measurements, and the output that produced the highest total of forced vital capacity (FVC) and FEV1 was selected for analysis. A bronchodilator test was not done in the assessment of lung function. Predicted values based on the European Respiratory Society criteria were used [19]. The spirometric parameters were measured as a percentage of the predicted values, and included FVC, FEV1, the ratio between them (FEV1/FVC), peak expiratory flow (PEF), and forced expiratory flow at 25–75% of the pulmonary volume (FEF25–75%).

A restrictive spirometric pattern was defined by FVC < 80% of the predicted value with a FEV1/FVC ratio ≥ 70%, with a flow–volume curve showing a convex pattern [20]. An abnormal FEV1 was defined as a value lower than 80% of that predicted. An obstructive spirometric pattern, a disproportionate reduction of maximal airflow in relation to the maximal volume that can be displaced from the lung, was also identified by a ratio FEV1/FVC <70% [21].

### 2.4. Statistical Analysis

The normal distribution of variables was evaluated using the Shapiro-Wilk test. Data were expressed either as the mean ± standard deviation (SD) or the absolute number (percentage). Comparisons between groups were performed using the Student’s t test for continuous variables, and the Pearson’s chi-squared for categorical variables. Fischer’s exact test was used when cells had expected values of zero. The relationship between continuous variables was assessed by the Pearson’s linear correlation test, in which coefficients of 0–0.19, 0.2–0.39, 0.4–0.59, 0.6–0.79, and 0.8–1.0 indicate very weak, weak, moderate, strong, and very strong correlations, respectively. Three multivariable logistic regression models for the presence of a restrictive spirometric pattern, an abnormal FEV1 (FEV1 < 80% of predicted), and an obstructive spirometric pattern for cohort development were done including the following confounding variables in the analysis: baseline clinical variables that could affect pulmonary function (age, sex, BMI, and smoking habit), variables associated with type 1 diabetes (HbA1c, presence of retinopathy or nephropathy, and time from diagnosis) and variables associated with lung volumes in univariate analysis (insulin dosage). Model calibration was assessed using the Hosmer–Lemeshow test of fit and the area under the receiver operating characteristic (ROC) curve. The accuracy of basal insulin therapy as a measurement of interest in discriminating diseased subjects (patients with any spirometric pattern) from cases without any spirometric pattern was evaluated using a univariate logistic regression to derive the ROC curve analysis. A complete sensitivity/specificity report and calculating Youden J statistic was used. All “*p*” values were based on a two-sided test of statistical significance. Significance was accepted at the level of *p* < 0.05. The statistical analyses were performed using SSPS statistical package (IBM SPSS Statistics for Windows, Version 20.0. Armonk, NY, USA).

## 3. Results

The main pulmonary variables of the study population are presented in Table 3. Patients with type 1 diabetes showed lower FVC (95.0 ± 11.9 vs. 99.7 ± 11.0% of predicted, *p* = 0.017), lower FEV1 (95.2 ± 12.8 vs. 100.2 ± 10.5% of predicted, *p* = 0.015) and PEF (91.7 ± 10.9 vs. 102.9 ± 16.4% of predicted, *p* = 0.030) in comparison to the control group. In addition, a higher prevalence of subjects with a restrictive ventilatory pattern (10.7% vs. 0%, *p* = 0.006) and FEV1 < 80% (10.7% vs. 2.7%, *p* = 0.044) were also present in patients with type 1 diabetes than in control subjects. However, no differences in the prevalence of an obstructive ventilatory pattern were observed between groups.

In the entire population of patients with type 1 diabetes, a greater prevalence of a restrictive ventilatory pattern (38.9% vs. 8.7%, *p* < 0.001) and abnormal FEV1 (44.4% vs. 9.4%, *p* < 0.001) appeared among those with the diagnosis of diabetic retinopathy in comparison to subjects free of this complication. Additionally, a higher percentage of subjects with an abnormal FEV1 pattern were observed among those patients with diabetic nephropathy (21.6% vs. 9.6%, *p* = 0.045). Regarding to the ischemic heart disease, no differences among respiratory patterns were observed.

**Figure 1:**
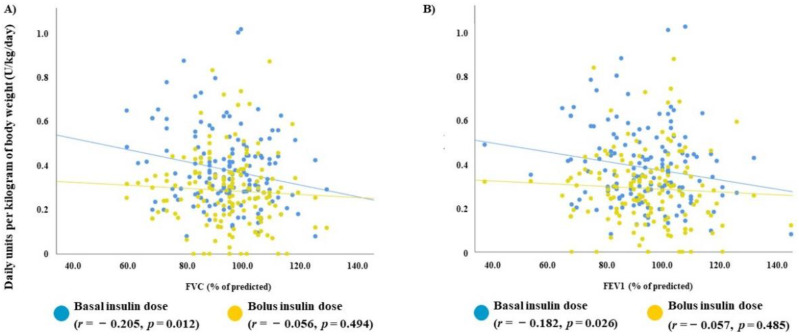
Scatter plot showing the linear correlation between pulmonary parameters (forced vital capacity and forced expiratory volume in the first second) and daily units per kilogram of body weight (U/kg/day) of basal (blue circle) and bolus (yellow circle) insulin. (**A**) FVC (% of predicted), (**B**) FEVI (% of predicted).

**Figure 2:**
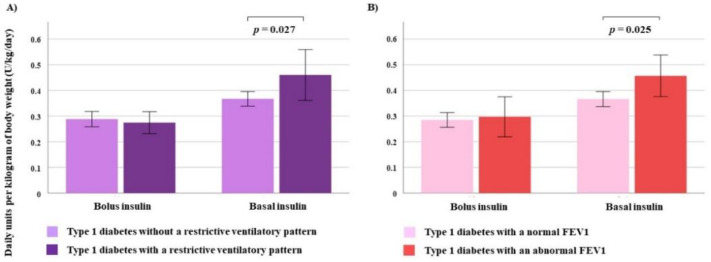
Plot showing the basal-bolus insulin therapy in patients with type 1 diabetes according to: (**A**) a restrictive ventilatory pattern and (**B**) an abnormal (<80% of predicted) forced expiratory volume in the first second.

In the univariate analysis, the dose of basal insulin (U/kg/day) showed a negative and significant correlation with FVC (*r* = −0.205, *p* = 0.012) and FEV1 (*r* = −0.182, *p* = 0.026). These correlations were classified as “weak” and “very weak,” respectively. This relationship disappeared when the dose of bolus insulin was evaluated (Figure 1). The other available spirometric parameters did not correlate with basal or bolus insulin therapy (Table 4). In parallel, patients with type 1 diabetes and a restrictive ventilatory pattern, as well as those with FEV1 < 80%, were treated with significantly higher doses of basal insulin in comparison to patients with a non-restrictive pattern (0.4 ± 0.2 vs. 0.3 ± 0.1 U/kg/day, *p* = 0.027) or FEV1 ≥ 80% (0.4 ± 0.1 vs. 0.3 ± 0.1 U/kg/day, *p* = 0.025) (Figure 2). However, no differences were detected in the daily dose of bolus insulin between patients with and without a restrictive pattern (0.2 ± 0.08 vs. 0.2 ± 0.1 U/kg/day, *p* = 0.735) or with and without a FEV < 80 (0.2 ± 0.1 vs. 0.2 ± 0.1 U/kg/day, *p* = 0.750).

According to ROC analysis, the optimal cut-off value for identifying patients with a restrictive spirometric pattern was 0.5 U/kg/day of basal insulin. At this point, the area under the ROC was 0.62 (0.47 to 0.77) with a sensitivity of 36.8% and a specificity of 90.1%. The ROC analysis also revealed that the best cut-off point for detecting patients with abnormal FEV1 was 0.4 U/kg/day of basal insulin. At this dosage, the area under the ROC was 0.65 (0.52 to 0.79) with a sensitivity of 66.7% and a specificity of 65.1%.

Finally, the multivariable logistic regression model exhibited that basal insulin (U/kg/day) (OR = 77.1 (3.2 to 1816.6), *p* = 0.007), but not years from date of diagnosis, the presence of microangiopathic complications, or data related with metabolic control, independently predicted the presence of a restrictive spirometric pattern (Table 5). In the same way, basal insulin (U/kg/day) appeared to be associated with FEV < 80% (OR = 29.9 (1.5 to 562.1), *p* = 0.023). In the third model, basal insulin (U/kg/day) was not associated with the presence of the obstructive spirometric pattern (*p* = 0.647).

## 4. Discussion

The current study reinforces the evidence that patients with type 1 diabetes suffer lung impairment in comparison to control subjects. This dysfunction is characterized by a restrictive spirometric pattern and appears to be related to higher doses of subcutaneous basal insulin. Moreover, respiratory patterns were associated with the presence of diabetic microangiopathy. However, the deleterious impact on pulmonary function is not related with metabolic control unlike in the case of those patients with type 2 diabetes [22]. As far as we know, no human studies linking subcutaneous insulin treatment and pulmonary dysfunction in type 1 diabetes have been published.

The prevalence of self-reported respiratory diseases in patients with type 1 diabetes (emphysema, chronic obstructive pulmonary disease, chronic bronchitis, and asthma) has been established to be of 26%, with an odds ratio of 1.62 (CI: 1.36–1.93) developing lung-related complications, compared to those subjects without diabetes [23]. In 1984, Schnapf et al. demonstrated the presence of a restrictive spirometric pattern in 21 patients with insulin-dependent diabetes mellitus and severe limited joint mobility, suggesting that this could be due to decreased lung compliance or restriction of chest wall expansion [24]. Similarly, results from the Study of Health in Pomerania also described a restrictive type of lung disease in 73 patients with type 1 diabetes, pointing to widespread collagen and elastin abnormalities as a main etiopathogenic mechanism [25]. In this way, skin autofluorescence, -a surrogate measurement of advanced glycation end products, has been related to a significant decrease in FVC and FEV1 values in subjects with prediabetes and type 2 diabetes [8].

Previous studies in adult populations have also demonstrated a reduction of pulmonary diffusion capacity for carbon monoxide (DLCO) in patients with type 1 diabetes that could be ascribed to a lower pulmonary capillary blood volume and an impairment in the regulation of pulmonary blood flow at the microvascular level [26]. In fact, DLCO was decreased in patients with type 1 diabetes under standard versus intensive treatment, in patients with complications in comparison with those without complications, and in patients with cardiac autonomic nervous system dysfunction versus those without it [27]. In addition, microcirculatory damage has also been shown to contribute to the depressed central chemosensitivity to hypercapnia described in patients with type 1 diabetes [28]. Moreover, a significant decrease in DLCO and reductions in FVC and FEV1 measurements has been associated with renal disease in patients with type 1 diabetes suggesting the same mechanism of action for both complications [16]. Our data confirms the relationship between renal and lung disease in patients with type 1 diabetes, but also adds a new association between the spirometric respiratory pattern and retinopathy that has not been previously recognized.

Our results failed to find a significant impact of standard glycemic control, measured by HbA1c, in the pulmonary function of patients with type 1 diabetes This result contradicts the results of Schnack et al. who described a clear correlation of pulmonary function tests with HbA1c measurements in 39 patients with long-standing type 1 diabetes [16]. Similarly, in children with type 1 diabetes inconsistent results have been obtained, with some studies favoring the role of metabolic control on pulmonary function and others showing an absolute lack of association between them [9,25].

A deleterious effect of insulin therapy has been earlier related with short-acting human insulin powder taken by oral inhalation [12,13,14]. Technosphere insulin is associated with lower risk of hypoglycemia and weight gain compared with insulin aspart, but dry cough 10 min after inhalation has been described by 24–33% of patients. Inhaled insulin also shows small declines from baseline in all parameters of pulmonary function within the first 3 months that resolve 4 weeks after discontinuation [12,13,14]. In this way, a decline equal or higher than ≥15% occurred in 6% of Technosphere insulin treated subjects compared to 3% of comparator-treated subjects [13,14]. The exact mechanisms of lung dysfunction after insulin inhalation are unclear, but animal studies described the formation of amyloid aggregates and the induction of mitochondrial dysfunction leading to a significant impairment in pulmonary air flow [29].

Apart from a direct effect of insulin on lung function, our data also point to higher doses of long-acting subcutaneous insulin as one of the factors may be related to this deleterious outcome. In the Copenhagen City Heart Study, which comprised 68 patients with type 1 diabetes and 323 patients with type 2 diabetes, pulmonary injury assessed through FEV1 and FVC was somewhat more pronounced in those treated with insulin in comparison with those treated with diet or oral agents [30]. However, this finding was attributed to the severity and duration of diabetes rather than to the insulin itself. The hyperinsulinemic consequences in airway structure and function engage the increased proliferation of primary human airway smooth muscle cells, the induction of collagen release and deposition, and a significant increase in airway hyperresponsiveness. These actions are mediated by the activation of the PI3/Akt-β-catenin axis which in an insulin-dependent manner leads to a proconstrictive and profibrotic phenotype, developing combined changes between restrictive and obstructive patterns of pulmonary function [11]. Our study identified the daily dose of basal insulin, but not of bolus insulin, as an independent factor for the presence of both a restrictive spirometric pattern and abnormal FEV1, suggesting that only the pharmacologic steady-state accumulation of insulin favors the injurious effect on the lung. Furthermore, the best cut-off points for detecting patients with a restrictive spirometric pattern and an abnormal FEV1 were 0.5 and 0.4 U/kg/day of basal insulin, respectively. However, it should not be forgotten that the established correlation between basal insulin dose and lung function in our study should be classified as weak or very weak. The association between type 1 diabetes and lung involvement is not a novelty [21,31]. In a population-based patient-centric data from The Netherlands, the use of asthma medication during the first 5 years after the diagnosis of type 1 diabetes was significantly higher than in a reference cohort with the same age and gender (23.2% vs. 18.3%) after the onset of diabetes [31]. In a cross-sectional study that included 196 patients with type 2 diabetes, Vargas et al. compared lung function between those receiving metformin or insulin secretagogues [32]. After adjustment for metabolic control and the duration of the disease, patients under treatment with therapies favoring hyperinsulinemia showed significantly higher differences from the expected values of FVC compared with those treated with metformin. In addition, as type II alveolar cells express insulin receptors that favor surfactant synthesis, the role of insulin resistance in initiating lung abnormalities also needs to be considered [33]. The measurement of daily insulin dose as a ratio per body weight may be a better indicator of insulin resistance than other biomarkers such as BMI, waist circumference of total daily insulin dose. In addition, the estimated visceral adiposity was not related with pulmonary function parameters in our cohort. In obese women without diabetes insulin resistance was recognized as an independent predictor of altered airway resistance [34]. Overweightness and obesity continue to be prevalent among individuals with type 1 diabetes and emerging evidence suggests that obesity contributes to insulin resistance and is a catalyst for cardiometabolic complications in type 1 diabetes [10].

The long-term clinical sequels of the slight decrease in lung function for our population of patients with type 1 diabetes needs to be elucidated. Nevertheless, similar decrements of FEV1 values have been identified as an independent risk factor for all-cause mortality after only 7-years follow-up in patients with type 2 diabetes from the Fremantle Diabetes Study [35].

This study has some limitations that need to be considered. Certainly, the cross-sectional nature of the study does not allow us to establish causality. However, our results point to the lung function impairment as an unsuspected complication in patients with type 1 diabetes, especially in those who need higher doses of basal insulin. Second, we have no direct measures of insulin resistance to assert the association of this condition with lung dysfunction in type 1 diabetes. Third, although the sample size is relatively small, our study collects lung function data in the largest cohort of patients with type 1 diabetes evaluated so far.

## 5. Conclusions

In summary, patients with type 1 diabetes display a slight reduction in lung function measurements. Basal, but not bolus, insulin dose according to body weight seems to be related with the worst lung function present in this population. More in-depth studies are needed to confirm the deleterious effect and to explore the potential mechanisms implicated in the negative impact of subcutaneous basal insulin on the lung.

## Figures and Tables

**Table 1:** Main clinical and metabolic characteristics of participants in the case-control study according to the presence of type 1 diabetes.

	Type 1 Diabetes (*n* = 75)	Non-Type 1 Diabetes (*n* = 75)	Mean Difference (95% CI)	*p*
Age (years)	40.7 ± 12.6	40.2 ± 12.4	−0.5 (−4.6 to 3.5)	0.790
Women, *n* (%)	53 (71)	53 (71)	–	1.000
Body mass index (kg/m^2^)	24.6 ± 3.7	24.3 ± 3.6	−0.2 (−1.4 to 0.9)	0.632
Waist circumference (cm)	86.6 ± 12.5	89.5 ± 17.7	2.8 (−2.1 to 7.8)	0.262
Neck circumference (cm)	35.6 ± 3.4	36.0 ± 4.5	0.4 (−2.3 to 3.3)	0.742
Visceral adipose tissue (cm^2^)	94.6 ± 78.6	102.0 ± 88.6	7.3 (−19.6 to 34.4)	0.591
Lean body mass (kg)	46.8 ± 7.5	47.9 ± 6.1	1.1 (−1.0 to 3.4)	0.293
Former smoker, *n* (%)	13 (17)	10 (13)	–	0.820
Current smoker, *n* (%)	11 (15)	12 (16)	–	0.908
Fasting glucose (mmol/L)	8.8 ± 3.5	5.9 ± 0.7	−2.9 (−4.0 to −1.9)	<0.001
HbA1c (%)	7.6 ± 1.1	5.1 ± 0.4	−2.4 (−2.7 to −2.1)	<0.001
HbA1c (mmol/mol)	59.8 ± 12.4	33.0 ± 4.3	−26.7 (−29.7 to −23.7)	<0.001
HbA1c ≥7.0%, *n* (%)	52 (69.3)	–	-	-

Data are expressed as a mean ± SD or n (percentage). HbA1c: glycosylated hemoglobin. Patients with type 1 diabetes showed 17.8 ± 11.9 years from the diagnosis of the disease and were under treatment with 0.3 ± 0.1 U/kg/day of basal insulin and 0.2 ± 0.1 U/kg/day of bolus insulin. The prevalence of chronic complications in patients with type 1 diabetes was: 8.0% diabetic retinopathy, 28% diabetic nephropathy, and 2.6% had suffer an ischemic heart disease.

**Table 2:** Main clinical and metabolic characteristics of the entire population with type 1 diabetes in which the influence of insulin therapy on lung function was evaluated.

	Type 1 Diabetes (*n* = 150)
Age (years)	38.6 ± 14.9
Women, *n* (%)	67 (44.7)
Body mass index (kg/m^2^)	24.4 ± 3.8
Waist circumference (cm)	87.5 ± 12.9
Visceral adipose tissue (cm^2^)	101.3 ± 83.0
Lean body mass (kg)	49.2 ± 7.9
Former smoker, *n* (%)	16 (10.6)
Current smoker, *n* (%)	36 (24.0)
Fasting glucose (mmol/L)	9.9 ± 5.1
HbA1c (%)	8.0 ± 1.6
HbA1c (mmol/mol)	64.0 ± 17.8
HbA1c ≥7.0%, *n* (%)	107 (71.3)

Data are expressed as mean ± SD or n (percentage). HbA1c: glycosylated hemoglobin; BMI: body mass index. This population showed 17.2 ± 12.1 years from the diagnosis of the disease and were under treatment with 0.3 ± 0.1 U/kg/day of basal insulin and 0.2 ± 0.1 U/kg/day of bolus insulin. The prevalence of chronic complications in patients with type 1 diabetes was: 12.0% diabetic retinopathy, 34.0% diabetic nephropathy, and 4.6% had suffer an ischemic heart disease.

**Table 3:** Main pulmonary function variables and breathing patterns of the study population according to the presence of type 1 diabetes.

	Type 1 Diabetes (*n* = 75)	Non-Type 1 Diabetes (*n* = 75)	Mean Difference (95% CI)	*p*
FVC (% predicted)	95.0 ± 11.9	99.7 ± 11.0	−4.7 (−8.3 to −1.8)	0.017
FEV1 (% predicted)	95.2 ± 12.8	100.2 ± 10.5	−6.0 (−9.5 to −2.1)	0.015
FEV1/FVC	91.7 ± 11.0	85.1 ± 6.1	6.6 (2.9 to 8.7)	<0.001
PEF (% predicted)	91.7 ± 10.9	102.9 ± 16.4	−11.2 (−16.2 to −6.5)	0.030
FEF25–75% (% predicted)	87.1 ± 24.1	92.4 ± 23.0	−5.3 (−12.6 to 0.4)	0.288
Restrictive spirometric pattern, *n* (%)	8 (10.7)	0 (0)	–	0.006
FEV1 < 80%, *n* (%)	8 (10.7)	2 (2.7)	–	0.044
Obstructive spirometric pattern, *n* (%)	1 (1.3)	2 (2.7)	–	0.559

Data are expressed as mean ± SD or n (percentage). FVC: forced vital capacity; FEV1: forced expired volume in the first second; PEF: peak expiratory flow; FEF25–75%: forced expiratory flow at 25–75% of the pulmonary volume. Value expressed as a percentage of the predicted value.

**Table 4:** Bivariate correlation between pulmonary parameters and relevant medical information in patients with type 1 diabetes.

	FVC (% Predicted)		FEV1 (% Predicted)		PEF (% Predicted)		FEF 25–75 (% Predicted)	
	*r*	*p*	*r*	*p*	*r*	*p*	*r*	*p*
Age (years)	−0.042	0.608	−0.003	0.973	−0.076	0.354	−0.084	0.304
HbA1c (%)	−0.001	0.989	−0.034	0.683	−0.118	0.150	−0.035	0.670
Body mass index (kg/m^2^)	−0.098	0.231	0.019	0.819	0.169	0.039	0.154	0.061
Waist circumference (cm)	−0.311	0.130	−0.144	0.493	−0.177	0.397	0.051	0.808
Visceral AT (cm^2^)	−0.093	0.166	−0.049	0.466	0.036	0.596	−0.004	0.952
TDD of insulin (U/kg/day)	−0.157	0.055	−0.144	0.080	−0.067	0.413	−0.014	0.864
Basal insulin dose (U/kg/day)	−0.205	0.012	−0.182	0.026	−0.086	0.297	−0.023	0.778
Bolus insulin dose (U/kg/day)	−0.056	0.494	−0.057	0.485	−0.024	0.766	−0.004	0.959

TDD: total daily dose.

**Table 5:** A multivariable logistic regression model for the presence of a restrictive spirometric pattern and an abnormal (<80% of predicted) forced expiratory volume in the first second in patients with type 1 diabetes.

Restrictive Spirometric Pattern		OR (95% CI)	*p* Value
Age (years)		1.00 (0.95 to 1.05)	0.964
Years with type 1 diabetes		1.05 (0.99 to 1.11)	0.076
Sex	Women	Reference	
	Men	1.58 (0.48 to 5.21)	0.452
Body mass index (kg/m^2^)		1.00 (0.86 to 1.17)	0.992
HbA1c	<7.0%	Reference	
	≥7.0%	1.75 (0.47 to 6.54)	0.404
Basal insulin (U/kg/day)		77.14 (3.27 to 1816.63)	0.007
Bolus insulin (U/kg/day)		0.05 (0.00 to 2.56)	0.142
Smoking habit	Never	Reference	
	Current	2.56 (0.41 to 16.00)	0.315
	Former	0.34 (0.02 to 5.13)	0.439
Retinopathy	No	Reference	
	Yes	1.02 (0.28 to 3.68)	0.975
Nephropathy	No	Reference	
	Yes	1.04 (0.98 to 1.11)	0.725
Hosmer–Lemeshow test of fit			0.975
Area under the ROC curve		0.79 (0.69 to 0.90)	<0.001
**FEV1 < 80%**			
Age (years)		1.05 (1.00 to 1.09)	0.042
Years with type 1 diabetes		0.99 (0.95 to 1.04)	0.794
Sex	Women	Reference	
	Men	1.51 (0.52 to 4.38)	0.451
Body mass index (kg/m^2^)		1.02 (0.89 to 1.17)	0.741
HbA1c	<7.0%	Reference	
	≥7.0%	1.17 (0.36 to 3.75)	0.798
Basal insulin (U/kg/day)		29.93 (1.59 to 562.81)	0.023
Bolus insulin (U/kg/day)		0.74 (0.03 to 17.48)	0.854
Smoking habit	Never	Reference	
	Current	0.91 (0.22 to 3.82)	0.892
	Former	0.66 (0.10 to 4.23)	0.663
Retinopathy	No	Reference	
	Yes	1.81 (0.52 to 6.30)	0.654
Nephropathy	No	Reference	
	Yes	2.02 (0.60 to 6.84)	0.510
Hosmer–Lemeshow test of fit			0.195
Area under the ROC curve		0.73 (0.62 to 0.85)	<0.001

## Supplementary Materials

The following are available online at https://www.mdpi.com/2077-0383/9/5/1249/s1, Figure S1. Flow chart of the study population.
