# Supplementary figures and images for: Effect of Subcutaneous Insulin on Spirometric Maneuvers in Patients with Type 1 Diabetes: A Case-Control Study
Source: J Clin Med. 2020 Apr 25;9(5):1249. doi: 10.3390/jcm9051249 (PMC7287574; doi:10.3390/jcm9051249)

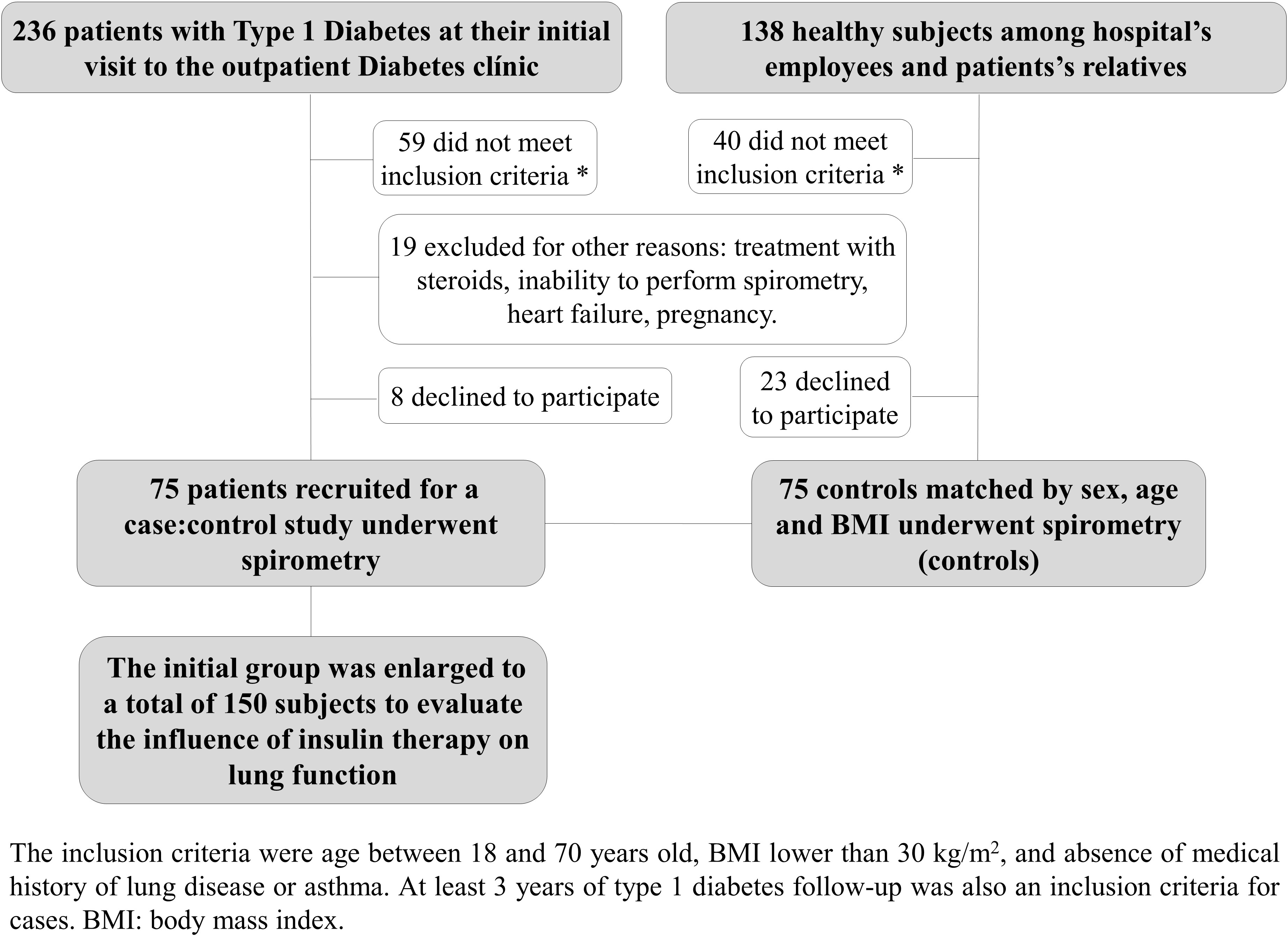

Supplement: Supplementary file 1 [file jcm-09-01249-s001.zip › jcm-771357-supplementary.jpg]
